# Clinical and Injury-Related Factors Associated with In-Hospital Complications After Traumatic Spinal Injury: A 7-Year Retrospective Cohort Study

**DOI:** 10.3390/jcm15155822

**Published:** 2026-07-25

**Authors:** Nizar Algarni, Khalid Alrasheed, Othman Alabdullah, Abdulaziz Almanea, Musab Alageel, Abdulrahman Alaseem, Yousef Marwan, Abdullah Addar

**Affiliations:** 1 Department of Orthopedic Surgery, College of Medicine, King Saud University, Riyadh 11451, Saudi Arabia; 2 College of Medicine, King Saud University, Riyadh 11451, Saudi Arabia; 3 Department of Surgery, College of Medicine, Health Sciences Center, Kuwait University, Kuwait City 13110, Kuwait

**Keywords:** traumatic spinal injury, in-hospital complications, polytrauma, trauma, spinal cord injury, spine, Saudi Arabia

## Abstract

**Background/Objectives**: Traumatic spinal injury (TSI) is associated with substantial morbidity, but factors contributing to in-hospital complications remain insufficiently defined. This study evaluated factors associated with in-hospital complications after TSI. **Methods**: We conducted a retrospective cohort study at a tertiary center (January 2018–May 2025). Among 5380 trauma patients screened by computed tomography, 413 admitted patients with TSI were included. The primary outcome was any documented in-hospital complication. Factors were assessed using multivariable logistic regression and reported as adjusted odds ratios (ORs) with 95% confidence intervals (CIs). **Results**: The median age was 28.0 years, 337 were male (81.6%), and four-wheel motorized vehicle accidents were the most common injury mechanism (57.1%). In-hospital complications occurred in 99 patients (24.0%). Pulmonary infection was most common (9.9%), followed by bloodstream infection (4.4%), urinary tract infection (3.9%), and surgical site infection (3.9%). Compared with patients without complications, those with complications had longer hospital stays, higher ICU admissions, longer ICU stays, and lower discharge home. In multivariate logistic regression, complications were associated with worse AIS grade (OR 1.690, 95% CI 1.241–2.302), inpatient physical therapy requirement (OR 4.385, 95% CI 2.494–7.711), greater number of associated non-spinal injuries (OR 1.729, 95% CI 1.463–2.042), and greater cervical vertebral injury burden (OR 1.479, 95% CI 1.061–2.062). **Conclusions**: Neurological severity, polytrauma burden, and cervical injury were independently associated with complications, supporting early risk stratification and multidisciplinary prevention in high-risk patients. Findings should be interpreted considering the retrospective single-center design, predominantly young male cohort, low spinal cord injury proportion, and limited post-discharge data.

## 1. Introduction

Traumatic spinal injury (TSI) is a subset of trauma encompassing structural injuries to the spinal column, including vertebral fractures, dislocations, and ligamentous injuries, with or without associated traumatic spinal cord injury (TSCI) [1]. TSI imposes a substantial global trauma burden, with an estimated incidence of 10.5 cases per 100,000 persons annually, roughly 768,000 new cases worldwide [2]. Road traffic collisions and falls are among the leading mechanisms of injury, although their relative contribution varies by region, socioeconomic development, and age distribution [2,3]. Beyond their immediate clinical impact, TSIs pose a substantial burden on healthcare systems, given the costly and complex medical care affected patients require, as well as the economic ramifications of lost productivity [4,5]. In Saudi Arabia, spinal trauma is particularly relevant given the historically high contribution of road traffic collisions to severe injury [6]. Local studies show it predominantly affects young male patients and is commonly associated with concomitant injuries, hospitalization, and adverse outcomes [7,8,9].

Although TSI and TSCI are related, they should not be used interchangeably. TSCI specifically refers to traumatic damage to the spinal cord resulting in motor, sensory, or autonomic dysfunction [10]. In contrast, TSI includes spinal-column injuries with or without TSCI [1]. This distinction is clinically relevant because management decisions in spinal trauma are guided not only by neurological deficit, but also by injury morphology and mechanical stability [11]. Accordingly, complication risk after TSI may reflect not only neurological impairment, but also the broader burden of high-energy trauma, associated injuries, immobilization, operative treatment, and intensive care unit (ICU) exposure. In-hospital complications following TSI represent a major determinant of patient outcomes and healthcare resource utilization. Patients with TSCI may develop respiratory infections, urinary tract infections, pressure ulcers, venous thromboembolism, wound-related complications, and other systemic adverse events during hospitalization [12,13]. Previous studies have demonstrated that factors such as advanced age, spinal cord injury, neurological deficits, injury severity, polytrauma, and delayed intervention may increase the risk of adverse in-hospital outcomes [14,15,16].

Despite the growing literature on complications after TSCI, less is known about factors associated with in-hospital complications across the broader spectrum of admitted TSI patients, including those with vertebral fractures or dislocations without spinal cord injury [17,18]. This gap is important because neurologically intact patients may still require immobilization, surgery, ICU admission, prolonged hospitalization, and treatment for associated non-spinal injuries. In Saudi Arabia, available studies have mainly described the epidemiology, mechanisms, and general outcomes of spinal trauma [7,8,9], while data specifically evaluating clinical and injury-related factors associated with in-hospital complications among admitted TSI patients remain limited.

Therefore, this retrospective cohort study aimed to evaluate the factors associated with the development of in-hospital complications among admitted patients with TSI treated at a tertiary care center in Riyadh, Saudi Arabia. Secondary objectives were to characterize the distribution of specific in-hospital complications and examine the association between complication development and hospitalization outcomes, including ICU utilization, hospital length of stay, discharge disposition, and in-hospital mortality.

## 2. Materials and Methods

### 2.1. Study Design and Setting

This retrospective cohort study was conducted at King Khalid University Hospital (KKUH) in Riyadh, Saudi Arabia. Riyadh is the capital city of Saudi Arabia and has a population of almost 8 million people. The study included trauma patients who presented to the emergency department between January 2018 and May 2025. The study was prepared in accordance with the Strengthening the Reporting of Observational Studies in Epidemiology (STROBE) guidelines [19].

### 2.2. Data Source and Case Identification

Potentially eligible patients were identified through a search of the KKUH electronic medical record (EMR). The search included all trauma patients who underwent whole-body or selective spine computed tomography (CT) scans during the study period. Imaging was performed according to institutional trauma protocols, and CT assessment was based on multidetector CT images with sagittal and coronal multiplanar reconstructions. These records were screened for spine injury, and patients with confirmed spine injury were identified. Their medical record numbers were then entered into a standardized data collection sheet for eligibility assessment and subsequent data extraction for outcome variables if they met the inclusion and exclusion criteria.

### 2.3. Participants

**Figure 1:**
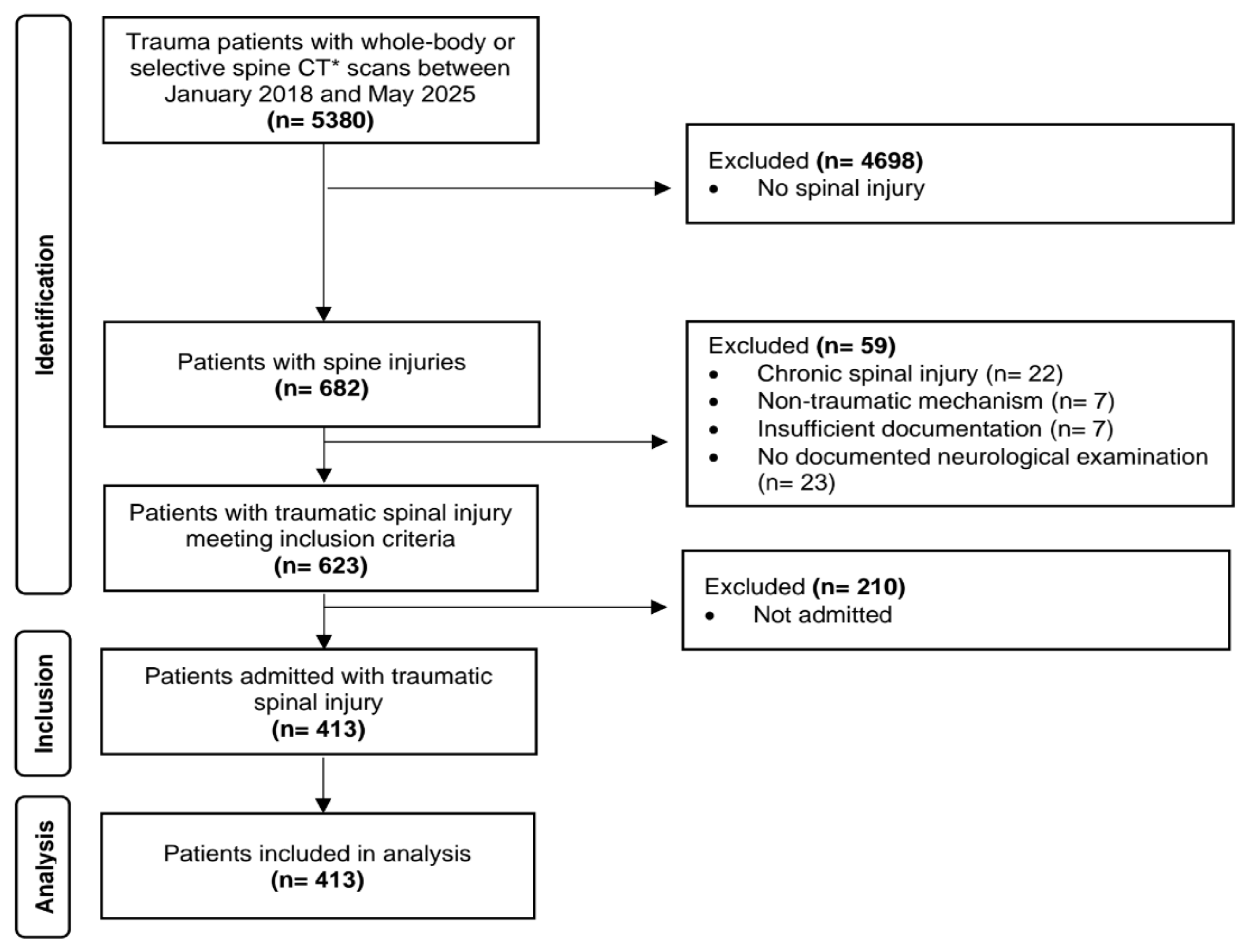
Patient selection flow diagram. CT*, computed tomography.

A total of 5380 trauma patients were identified through the EMR search. Of these, 4698 were excluded as no spinal injury was present, leaving 682 (12.7%) to be assessed for eligibility. Patients were eligible for the TSI cohort if they had an acute spine injury caused by a traumatic mechanism. TSI was defined as vertebral fracture, spinal subluxation, spinal dislocation, traumatic intervertebral disc herniation, or spinal cord injury at any spinal level. Patients with clinical manifestations suggestive of neurological deficit who were subsequently evaluated by MRI and diagnosed with spinal cord injury were also included. Patients with isolated spinal injury and those with polytrauma involving spinal injury were included. Fifty-nine were excluded because of chronic spinal injury, non-traumatic injury mechanism, insufficient documentation, or no documented neurological examination. This yielded 623 patients with acute TSI. Because the study focused on in-hospital complications, the cohort was restricted to admitted patients. After excluding 210 non-admitted patients, 413 were included in the final analysis. The full patient selection is summarized in Figure 1.

### 2.4. Variables

Variables of interest included demographics, injury mechanism, spinal injury characteristics, neurological status, associated injuries, hospital course, and final disposition. Demographic variables included age, sex, nationality, substance use at the time of injury, and prior psychiatric diagnosis. The injury mechanism included four-wheel motorized vehicle accidents (Four W-MVA, specifying driver/passenger status), two-wheel motorized vehicle accidents (Two W-MVA), pedestrian injuries, falls from the ground, falls from height, being struck by an object, assault, and sport/recreation. Neurological status was assessed using the American Spinal Injury Association Impairment Scale (AIS), with AIS grades A–E. AIS grade was recorded from the earliest documented neurological examination after initial trauma assessment during the index admission. Spinal injury characteristics included injury diagnosis and associated injury level. Associated injuries were recorded as the presence of any associated injury and by injury type during the index admission, when confirmed by clinical examination, imaging, operative findings, or clinical documentation. Injuries identified later were included if attributable to the index trauma event. Hospital-course variables, including operative treatment, hospital length of stay, ICU admission, ICU length of stay, inpatient physical therapy need, in-hospital mortality, and final disposition, were also collected. Receipt of inpatient physical therapy was recorded when a formal treatment session was documented during the index admission. Referral to physiotherapy was determined by the treating team and was influenced by hemodynamic stability, spine precautions, fracture stability, neurological status, operative timing, ICU status, weight-bearing restrictions, pain control, and overall functional limitation. When initiated, rehabilitation commonly included bed mobility, transfer training, when indicated, strengthening, gait training, and discharge planning according to clinical need.

### 2.5. Outcome Measures

The primary outcome was the development of any in-hospital complication among admitted patients with TSI. This was recorded as a binary outcome, defined as the presence of at least one documented complication during the index hospital admission. Individual complications were also recorded by type, including pulmonary infection, urinary tract infection, pressure ulcers/bedsores, venous thromboembolism, surgical site infection, bloodstream infection, meningitis, delirium, fever of unknown origin, electrolyte disturbance, acute kidney injury, and other documented hospital complications.

Infection types were defined using clinical documentation. Pulmonary infection was defined as documented pneumonia or lower respiratory tract infection supported by clinical assessment, radiographic findings, and microbiology when available. Bloodstream infection was defined as documented bacteremia or sepsis with positive blood culture and clinical treatment, excluding likely contaminants. Urinary tract infection was defined as a documented UTI supported by clinical assessment and urine testing. Surgical site infection was defined as documented wound or operative-site infection requiring antimicrobial therapy, drainage, debridement, or other treatment. Meningitis was defined as a documented clinical diagnosis supported by cerebrospinal fluid testing. A fever of unknown origin was recorded when the fever prompted an infectious evaluation without a confirmed source.

Infection categories were not mutually exclusive, meaning patients could have more than one infection type during the index admission. For descriptive reporting, each complication type was counted separately. For the multivariable analysis, the outcome remained binary at the patient level, defined as any documented in-hospital complication. Secondary outcomes included the distribution of individual complication types and the number of in-hospital complications per patient.

### 2.6. Statistical Analysis

Categorical variables were presented as frequencies and percentages, with a multiple-response dichotomy used for variables allowing more than one response. Continuous variables were presented as means and standard deviations, or medians and interquartile ranges (IQR) when they violated the assumption of statistical normality. The Kolmogorov–Smirnov statistical normality test and a histogram were used to assess the statistical normality assumption for metric variables. Baseline characteristics were compared between patients with and without in-hospital complications using the Mann–Whitney U test for continuous variables. Categorical variables were compared using Pearson’s chi-square test when expected cell counts were adequate and Fisher’s exact test when cell counts were small. For larger sparse contingency tables, Fisher’s exact test with Monte Carlo simulation using 10,000 replicates was used. A multivariable binary logistic regression analysis was conducted to identify clinical and demographic factors associated with in-hospital complications among TSI patients, where the dependent outcome variable was binary (indicating whether complications occurred during the treatment course). The association between independent variables and the development of complications was expressed as multivariable adjusted odds ratios (OR) with their associated 95% confidence intervals. AIS grades were reverse-coded so that higher values reflect worse neurological status (AIS grade A = 5, E = 1). The IBM SPSS statistical program version 30 (IBM Corp., Armonk, New York, USA) and R statistical software version 4.6.0 (R Foundation for Statistical Computing, Vienna, Austria) were used for statistical analysis. R statistical software was used to create figures. Statistical significance was defined as a *p*-value <0.050.

### 2.7. Ethical Considerations

All methods were conducted in accordance with the Declaration of Helsinki. The study proposal was approved by the King Saud University ethical review board (reference number: 25/0130/IRB; approval of date 13 February 2025). Given the retrospective design and use of existing medical records, informed consent was waived by the approving committee.

## 3. Results

### 3.1. Baseline and Injury Characteristics by In-Hospital Complication Status

Baseline and injury characteristics of the admitted cohort stratified by in-hospital complication are presented in Table 1. Among the admitted cohort, 99 patients (24.0%) developed at least one in-hospital complication, while 314 patients (76.0%) did not. Patients with complications were slightly older than those without complications [30.0 years (IQR, 25.0–39.0) vs. 28.0 years (IQR, 22.0–36.8); *p* = 0.045]. Sex, substance use at the time of injury, and prior psychiatric diagnosis did not differ significantly between groups. Saudi nationality was less frequent among patients with complications (58.6% vs. 71.7%; *p* = 0.015). Injury mechanism did not differ significantly between groups (*p* = 0.082). Four W-MVAs were the most common mechanism in both groups, although they were less frequent among patients with complications (47.5% vs. 60.2%). In contrast, pedestrian injuries were more common in the complication group (22.2% vs. 11.5%). Among patients involved in Four W-MVAs, the victim role differed significantly by complication status (*p* = 0.007), with a higher proportion of drivers and a lower proportion of passengers among patients with complications. Patients with complications had more severe injury patterns, including higher rates of spinal cord injury (13.1% vs. 1.9%; *p* < 0.001), cervical injury (31.3% vs. 21.0%; *p* = 0.035), thoracic injury (49.5% vs. 36.6%; *p* = 0.022), and AIS grades A–D (22.2% vs. 4.8%; *p* < 0.001).

### 3.2. Associated Injuries Among Admitted Patients with TSI Stratified by In-Hospital Complications

Associated injuries were more frequent and more extensive in the complication group. Patients with complications had a higher median number of associated injuries [4.0 (IQR, 3.0–5.0) vs. 2.0 (IQR, 1.0–3.0); *p* < 0.001], and the presence of any associated injury was more common (96.0% vs. 87.3%; *p* = 0.014). Lower limb fractures, pelvic fractures, craniocerebral injuries, maxillofacial injuries, rib fractures, intra-thoracic injuries, and intra-abdominal injuries were all significantly more frequent among patients with complications (Table 2).

### 3.3. Hospital Course and Outcomes of Admitted Patients with TSI Stratified by In-Hospital Complications

Hospital course differed markedly between groups. Patients with complications were more likely to undergo surgery for non-TSIs (74.7% vs. 42.4%; *p* < 0.001), had longer hospital length of stay [37.0 days (IQR, 22.0–76.0) vs. 6.0 days (IQR, 3.0–12.0); *p* < 0.001], and had longer ICU length of stay [14.0 days (IQR, 6.0–21.0) vs. 3.0 days (IQR, 1.0–6.0); *p* < 0.001]. ICU admission (91.9% vs. 36.0%; *p* < 0.001) and receipt of inpatient physical therapy (70.7% vs. 31.5%; *p* < 0.001) were also more frequent in the complication group. Final disposition differed significantly between groups (*p* = 0.009), with lower discharge home and higher mortality among patients with complications (Table 3).

### 3.4. Frequency of In-Hospital Complications

**Figure 2:**
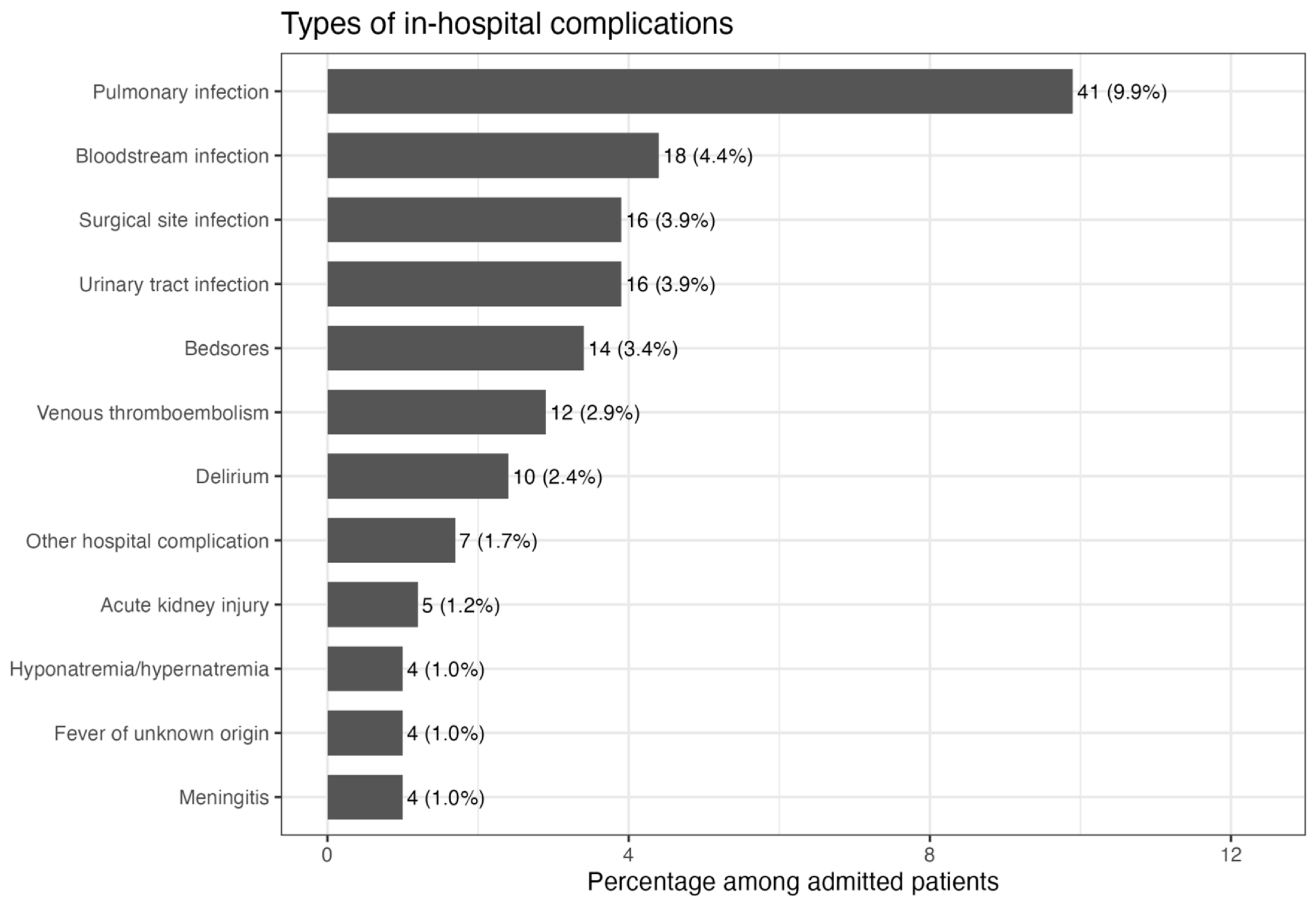
Frequency and distribution of in-hospital complications among admitted patients (*n* = 413).

Pulmonary infection was the most common complication (*n* = 41, 9.9%), followed by bloodstream infection (*n* = 18, 4.4%), urinary tract infection and surgical site infection (*n* = 16 each, 3.9%), bedsores (*n* = 14, 3.4%), venous thromboembolism (*n* = 12, 2.9%), and delirium (*n* = 10, 2.4%). Among patients with complications, most had one complication (62.6%), while 37.4% experienced two or more (Figure 2).

### 3.5. Factors Associated with In-Hospital Complications

Several variables were significantly associated with increased odds of experiencing in-hospital complications. Patients with higher AIS scores had significantly elevated odds of developing complications (OR = 1.690, 95% CI [1.241, 2.302], *p* = 0.001). Similarly, the need for inpatient physical therapy emerged as an independent factor (OR = 4.385, 95% CI [2.494, 7.711], *p* < 0.001). The number of non-spinal associated injuries was also positively associated with the risk of complications (OR = 1.729, 95% CI [1.463, 2.042], *p* < 0.001). Furthermore, a greater number of cervical vertebral injuries was significantly associated with higher complication risk (OR = 1.479, 95% CI [1.061, 2.062], *p* = 0.021). Although not statistically significant, passenger-related Four-wheel motorized vehicle accidents showed a protective trend (OR = 0.427, *p* = 0.064). Age, sex, nationality, and lumbar injury burden were not independently associated with the development of in-hospital complications in this model. The full set of adjusted odds ratios are presented in Table 4.

## 4. Discussion

In this retrospective cohort of admitted patients with TSI, in-hospital complications occurred in approximately one-quarter of cases and were associated with substantially greater ICU utilization, prolonged hospitalization, reduced discharge home, and higher mortality. The main factors independently associated with complications were worse AIS grade, greater associated injury burden, cervical injury burden, and requirement for inpatient physical therapy. These findings suggest that neurological severity and overall trauma burden, rather than spinal injury alone, are determinants of adverse in-hospital outcomes after TSI.

The complication rate observed in this study confirms that admitted patients with TSI represent a clinically vulnerable group, even when most injuries are vertebral fractures without documented TSCI. Prior studies in TSCI have reported a broad range of acute complications, including respiratory, infectious, thromboembolic, cardiac, genitourinary, skin-related, and other systemic events [14,15,20]. In a prospective multicenter cohort of patients with acute TSCI, complications were frequent during the initial hospitalization and were more common among patients with more severe AIS grades [14]. More recent registry-based data have also shown that major adverse events and immobility-related complications, such as intubation, unplanned ICU admission, venous thromboembolism, pressure ulcers, surgical site infection, and catheter-associated urinary tract infection, remain important during hospitalization after TSCI [15]. Comparisons with the present study should be made cautiously, however, because most previous reports focused on TSCI, whereas our cohort included the wider spectrum of admitted TSI patients, including neurologically intact vertebral fractures. This difference in case definition may help explain the lower overall complication rate in our cohort compared with TSCI-specific studies [14,15].

Recent TSI and spine-trauma studies provide additional context for interpreting our findings. Takoutsing et al. reported a substantially higher in-hospital complication rate of 52.9% in a low-resource TSI cohort, with pressure ulcers and paralytic ileus as the most frequent complications, and identified older age and delayed spine surgery as factors associated with in-hospital complications [17]. In contrast, pulmonary infection was the most common complication in our cohort, and the factors associated with in-hospital complications were worse AIS grade, greater associated injury burden, cervical injury burden, and inpatient physical therapy. These differences may reflect variation in healthcare setting, case mix, resource availability, timing of surgery, nursing care, rehabilitation access, and complication definitions. Porto et al., in a large United States nationwide cohort of patients with traumatic spinal vertebral fractures, also showed that complication risk after spine trauma is influenced by patient vulnerability and injury severity, including frailty, lower GCS, higher ISS, concomitant TSCI, and surgical intervention [18]. Together, these studies support the interpretation that in-hospital complications after TSI are multifactorial and depend on neurological severity, systemic trauma burden, baseline vulnerability, and healthcare-system factors [17,18].

Pulmonary infection was the most frequent complication in the present cohort. This is consistent with prior literature showing that respiratory complications are an important source of morbidity after TSCI, particularly in patients with cervical or high thoracic involvement [14,20,21]. In the prospective multicenter cohort by Grossman et al., respiratory failure and pneumonia were among the most frequent severe and moderate complications, and most complications occurred early after injury [14]. Cervical TSCI can compromise ventilation by weakening respiratory muscles, reducing lung expansion, and limiting cough effectiveness, thereby increasing susceptibility to atelectasis and infection [21]. In addition, acute TSCI may increase vulnerability to infection through factors such as intubation, impaired clearance of airway secretions, reduced mobility, nutritional compromise, and extensive operative treatment [22]. The predominance of pulmonary infection in our cohort, therefore, supports early respiratory risk assessment, pulmonary hygiene, mobilization when feasible, and close monitoring of patients with cervical injury or neurological deficit [14,20,21,22].

A worse AIS grade was independently associated with in-hospital complications. This finding is consistent with previous evidence showing that neurological severity is closely linked to complication risk after TSCI [14,15,18,20]. Patients with greater neurological impairment are more likely to have reduced mobility, impaired respiratory mechanics, bladder dysfunction, sensory loss, autonomic disturbance, and prolonged need for intensive monitoring or ICU-level care. These factors may increase the likelihood of pulmonary infection, urinary tract infection, pressure injury, venous thromboembolism, and systemic infection [14,15,20,21]. Accordingly, neurological assessment at presentation may help identify patients who require closer surveillance and more intensive preventive measures during admission.

The number of associated non-spinal injuries was also strongly associated with complications. Patients who developed complications had a higher burden of associated injuries, including pelvic fractures, craniocerebral injuries, rib fractures, intra-thoracic injuries, intra-abdominal injuries, and lower limb fractures. This suggests that in-hospital complications among patients with TSI may reflect the combined effect of spinal trauma and overall injury burden. This interpretation is supported by spine-trauma literature showing that markers of injury severity, including lower GCS and higher ISS, are associated with higher complication risk after traumatic spinal fractures [18]. Similarly, TSCI registry data show that more severe injury characteristics are associated with higher odds of both major adverse events and immobility-related complications [15]. These findings highlight the importance of early multidisciplinary trauma care involving spine surgery, orthopedic surgery, neurosurgery, critical care, rehabilitation, nursing, respiratory therapy, and infection-prevention teams [17].

Cervical injury burden was independently associated with increased odds of complications, whereas lumbar injury burden was not. This association is clinically plausible because cervical injuries, especially when accompanied by neurological impairment, can affect respiratory function, cough strength, autonomic regulation, and mobility [20,21]. Registry-based TSCI data have also reported a greater incidence of in-hospital adverse events among patients with cervical TSCI [15]. Nevertheless, cervical injury burden should be interpreted as a marker of higher-risk injury patterns rather than as direct evidence that cervical trauma alone causes complications. Cervical injuries may coexist with greater neurological severity and may require more intensive monitoring, airway assessment, immobilization, or operative management. These findings suggest that cervical involvement should be considered when planning respiratory monitoring, early rehabilitation, and complication-prevention strategies for admitted patients with TSI.

The association between inpatient physical therapy requirement and complications should be interpreted cautiously. Physical therapy is not a fixed baseline exposure and should not be interpreted as causing complications. Rather, receipt of inpatient physical therapy likely reflects the patient’s evolving clinical status, functional limitation, neurological impairment, spine precautions, ICU exposure, operative timing, immobility, and prolonged admission. Patients with longer admissions also have more time to both receive physical therapy and develop documented complications. Therefore, inpatient physical therapy requirement is best interpreted as a marker of rehabilitation need and clinical complexity rather than as a causal exposure. Future studies should examine the timing, intensity, and goals of rehabilitation to determine whether early structured rehabilitation can reduce complications or whether the physical therapy requirement primarily reflects underlying injury severity and system-level factors.

Patients with complications had worse hospitalization outcomes, including longer hospital stay, longer ICU stay, higher ICU admission, lower discharge home, and higher mortality. These associations are clinically expected, as complications can delay recovery, increase monitoring and treatment needs, and postpone discharge or rehabilitation transfer. However, the relationship between complications and length of stay is likely bidirectional: complications may prolong hospitalization, while prolonged hospitalization also increases the opportunity for complications to occur and be documented. Prior TSCI evidence also supports the clinical importance of acute infectious complications, as pneumonia, wound infection, and sepsis during acute admission have been associated with worse functional outcomes after TSCI [22].

The high ICU admission rate in the present cohort (49.4%) alongside low in-hospital mortality (1.5%) should be interpreted in the context of the young age distribution, low proportion of documented TSCI, and high burden of associated traumatic injuries. ICU admission may reflect polytrauma monitoring, perioperative care, associated injuries, airway or respiratory observation, and institutional trauma pathways rather than life-threatening neurological injury alone. Similarly, the overall median hospital length of stay of 9 days reflects the entire admitted cohort, whereas patients with complications had a substantially longer median stay than those without complications (37.0 vs. 6.0 days). These findings highlight the resource burden associated with complications and support the need for early risk stratification and preventive care.

In the Saudi context, the findings are particularly relevant because TSI remains closely linked to road traffic injuries and high-energy mechanisms [7,8,9]. Although mechanism of injury was not independently associated with complications in the adjusted model, Four-W MVA was the most common mechanism overall, and pedestrian injuries were relatively more frequent among patients who developed complications. These patterns reinforce the importance of road safety, injury prevention, and trauma-system optimization. From a hospital-care perspective, however, the present findings suggest that complication prevention should be guided more by neurological severity, cervical injury involvement, and associated injury burden than by mechanism alone. Although age, sex, and nationality were included as standard demographic covariates, they were not independently associated with in-hospital complications in the adjusted model. These findings should be interpreted in the context of a predominantly young, male, regionally based cohort, which is consistent with the epidemiology of TSI in Saudi Arabia.

This study has several strengths. It provides a detailed single-center clinical characterization of admitted patients with TSI over a seven-year period, including comprehensive neurological, injury-related, and hospital-course variables. By evaluating a broad spectrum of variables, the study provides clinically relevant insights into factors associated with in-hospital complications in patients with TSI. Its regional relevance also contributes to the limited literature on in-hospital complications following spinal trauma in Saudi Arabia.

Nevertheless, several limitations should be acknowledged. First, the retrospective single-center design may limit generalizability and is subject to documentation and coding bias. Because complications were identified from medical records, less severe or inconsistently documented events may have been missed, potentially underestimating the true complication rate. Second, the cohort was predominantly young and male, and only a relatively small proportion of patients had documented spinal cord injury, limiting the applicability of the findings to older populations, more balanced sex distributions, and TSCI-specific cohorts. Third, longer hospitalization may have increased the likelihood of detecting complications, which could overestimate the association between complications and length of stay. Fourth, some potentially relevant variables, including frailty, detailed injury severity measures, mechanical ventilation, timing of complication onset, standardized rehabilitation timing, post-discharge rehabilitation, vertebral artery injury, and ligamentous injury, were not consistently available. Vascular imaging was performed according to clinical indication rather than as a standardized screening protocol, so occult vertebral artery injury may have been underrecognized. Management-related data were limited to whether patients underwent surgery for spinal or non-spinal injuries. Details on nonoperative care and specific surgical techniques were not collected as study variables. Other allied-health interventions, including occupational therapy, psychology, speech and language therapy, and respiratory therapy, were also not consistently captured as structured variables. Discharge disposition was recorded from the medical record and should not be interpreted as functional independence or home suitability. Home-environment assessment, caregiver readiness, and mobility status at discharge were not consistently available. Finally, the study assessed in-hospital outcomes only and did not evaluate long-term neurological recovery, functional status, readmission, or post-discharge complications. Because the study period spanned pre-pandemic, COVID-19 pandemic, and post-pandemic years, calendar-period effects may have influenced admission volume, case-mix, ICU use, rehabilitation access, discharge disposition, and infection rates. As no formal time-period analysis was performed, residual confounding related to pandemic-era changes in care delivery cannot be excluded.

Future research should prioritize prospective multicenter studies using standardized definitions for in-hospital complications after TSI. These studies should record the timing of complication onset, injury severity measures, frailty or physiological reserve, mechanical ventilation, operative timing, ICU interventions, rehabilitation timing, allied-health involvement, discharge planning processes, and calendar-period or health-system variables. Incorporating these variables would help distinguish baseline risk factors from hospital-course markers and would clarify whether complications are preventable, consequences of injury severity, or partly related to care processes. Further studies should also assess post-discharge outcomes, including neurological recovery, functional independence, readmission, delayed complications, and long-term rehabilitation needs. Linking acute hospital data with rehabilitation and follow-up outcomes would provide a more complete understanding of the burden of TSI and may help identify modifiable factors that improve both short-term safety and long-term recovery.

## 5. Conclusions

In this single-center retrospective cohort of admitted patients with TSI, in-hospital complications occurred in 24.0% of cases and were associated with worse AIS grade, greater associated injury burden, and cervical injury burden. Patients with complications had longer hospital and ICU stays, greater ICU utilization, lower home discharge rates, and higher mortality. These findings support early risk stratification and targeted complication-prevention strategies, including respiratory monitoring, pulmonary hygiene, infection surveillance, pressure-injury prevention, venous thromboembolism prophylaxis, and multidisciplinary rehabilitation planning for high-risk patients. Interpretation should consider the retrospective single-center design, young male-predominant cohort, lack of standardized rehabilitation timing and post-discharge functional data, and the inability to formally assess calendar-period effects. The small TSCI subgroup, lack of systematic vertebral artery injury screening, and absence of standardized mobility and post-discharge rehabilitation data further limit subgroup-specific and functional interpretations.

## Figures and Tables

**Table 1:** Baseline and injury characteristics of admitted patients with TSI stratified by in-hospital complication status.

Variable	Overall (N = 413)	No Complication (N = 314)	Any Complication (N = 99)	*p*-Value
Median age, years (IQR)	28.0 (23.0–38.0)	28.0 (22.0–36.8)	30.0 (25.0–39.0)	0.045
Male sex (%)	337 (81.6)	252 (80.3)	85 (85.9)	0.210
Saudi Nationality (%)	283 (68.5)	225 (71.7)	58 (58.6)	0.015
Substance use at the time of injury (%)	35 (8.5)	25 (8.0)	10 (10.1)	0.505
Prior diagnosis of a psychiatric condition (%)	24 (5.8)	18 (5.7)	6 (6.1)	0.903
Mechanism of injury				
Four W-MVA (%)	236 (57.1)	189 (60.2)	47 (47.5)	0.082
Two W-MVA (%)	23 (5.6)	19 (6.1)	4 (4.0)	
Pedestrian (%)	58 (14.0)	36 (11.5)	22 (22.2)	
Fall from the ground (%)	11 (2.7)	9 (2.9)	2 (2.0)	
Fall from height (%)	74 (17.9)	53 (16.9)	21 (21.2)	
Struck by an object (%)	7 (1.7)	4 (1.3)	3 (3.0)	
Sport/Recreation (%)	3 (0.7)	3 (1.0)	0 (0.0)	
Assault (%)	1 (0.2)	1 (0.3)	0 (0.0)	
Four W-MVA victim role (*n* = 236)				
Driver (%)	146 (61.9)	113 (59.8)	33 (70.2)	0.007
Passenger (%)	76 (32.2)	68 (36.0)	8 (17.0)	
Unspecified (%)	14 (5.9)	8 (4.2)	6 (12.8)	
Injury diagnosis				
Spinal fracture (%)	411 (99.5)	313 (99.7)	98 (99.0)	0.422
Spinal cord injury (%)	19 (4.6)	6 (1.9)	13 (13.1)	<0.001
Spinal subluxation/dislocation (%)	17 (4.1)	13 (4.1)	4 (4.0)	1.000
Intervertebral disc injury (%)	4 (1.0)	2 (0.6)	2 (2.0)	0.244
Injury Level				
Occipital condyle (%)	28 (6.8)	19 (6.1)	9 (9.1)	0.294
Cervical (%)	97 (23.5)	66 (21.0)	31 (31.3)	0.035
Thoracic (%)	164 (39.7)	115 (36.6)	49 (49.5)	0.022
Lumbar (%)	220 (53.3)	162 (51.6)	58 (58.6)	0.224
Sacral (%)	73 (17.7)	52 (16.6)	21 (21.2)	0.290
Coccyx (%)	7 (1.7)	5 (1.6)	2 (2.0)	0.675
AIS, A-D (%)	37 (9.0)	15 (4.8)	22 (22.2)	<0.001
AIS				
A (%)	14 (3.4)	5 (1.6)	9 (9.1)	<0.001
B (%)	3 (0.7)	2 (0.6)	1 (1.0)	
C (%)	4 (1.0)	2 (0.6)	2 (2.0)	
D (%)	16 (3.9)	6 (1.9)	10 (10.1)	
E (%)	376 (91.0)	299 (95.2)	77 (77.8)	

AIS, American Spinal Injury Association impairment scale; Four W-MVA, Four-wheel motorized vehicle accidents; Two W-MVA, Two-wheel motorized vehicle accidents.

**Table 2:** Associated injuries among admitted patients with TSI stratified by in-hospital complication status.

Variable	Overall (N = 413)	No Complication (N = 314)	Any Complication (N = 99)	*p*-Value
Median number of associated injuries (IQR)	2.0 (1.0–4.0)	2.0 (1.0–3.0)	4.0 (3.0–5.0)	<0.001
Any associated injury (%)	369 (89.3)	274 (87.3)	95 (96.0)	0.014
Upper limb fractures (%)	143 (34.6)	104 (33.1)	39 (39.4)	0.253
Lower limb fractures (%)	124 (30.0)	86 (27.4)	38 (38.4)	0.037
Pelvic fractures (%)	111 (26.9)	70 (22.3)	41 (41.4)	<0.001
Upper/lower limb joint dislocations (%)	19 (4.6)	11 (3.5)	8 (8.1)	0.058
Craniocerebral injuries (%)	136 (32.9)	75 (23.9)	61 (61.6)	<0.001
Maxillofacial injuries (%)	110 (26.6)	76 (24.2)	34 (34.3)	0.047
Rib fractures (%)	156 (37.8)	105 (33.4)	51 (51.5)	0.001
Intra-thoracic injuries (%)	173 (41.9)	114 (36.3)	59 (59.6)	<0.001
Intra-abdominal injuries (%)	93 (22.5)	57 (18.2)	36 (36.4)	<0.001
Vascular injuries (%)	19 (4.6)	11 (3.5)	8 (8.1)	0.058
Non-spinal neurological injuries (%)	7 (1.7)	5 (1.6)	2 (2.0)	0.675
Amputations (%)	3 (0.7)	3 (1.0)	0 (0.0)	1.000

**Table 3:** Hospital course and outcomes of admitted patients with TSI stratified by in-hospital complication status.

Variable	Overall (N = 413)	No Complication (N = 314)	Any Complication (N = 99)	*p*-Value
Hospital course				
Operated on for spinal injury (%)	79 (19.1)	54 (17.2)	25 (25.3)	0.076
Operated on for non-spinal injury (%)	207 (50.1)	133 (42.4)	74 (74.7)	<0.001
Median hospital length of stay, days (IQR)	9.0 (4.0–22.2)	6.0 (3.0–12.0)	37.0 (22.0–76.0)	<0.001
ICU admission (%)	204 (49.4)	113 (36.0)	91 (91.9)	<0.001
Median ICU length of stay, days (IQR)	6.0 (2.0–14.3)	3.0 (1.0–6.0)	14.0 (6.0–21.0)	<0.001
Received inpatient physical therapy (%)	169 (40.9)	99 (31.5)	70 (70.7)	<0.001
Final disposition				
Mortality (%)	6 (1.5)	2 (0.6)	4 (4.0)	0.009
Home (%)	364 (88.1)	285 (90.8)	79 (79.8)	
Subacute rehab (%)	30 (7.3)	18 (5.7)	12 (12.1)	
Long-term facility (%)	13 (3.1)	9 (2.9)	4 (4.0)	

ICU, intensive care unit.

**Table 4:** Multivariable binary logistic regression analysis of patients’ odds of having in-hospital complications after TSI.

Variable	Multivariable Adjusted Odds Ratio (OR)	95% C.I. for OR		*p*-Value
		Lower	Upper	
Sex: Male	0.836	0.396	1.761	0.637
Age (years)	1.013	0.994	1.031	0.176
Nationality: Saudi	1.031	0.576	1.844	0.918
AIS neuropathy severity level	1.690	1.241	2.302	0.001
Requirement for inpatient physical therapy	4.385	2.494	7.711	<0.001
Role of the victim in the Four W-MVA: Passenger	0.427	0.173	1.050	0.064
Number of other associated injuries	1.729	1.463	2.042	<0.001
Number of injured cervical vertebrae	1.479	1.061	2.062	0.021
Number of injured lumbar vertebrae	1.166	0.974	1.396	0.094
Constant	0.007			<0.001

Dependent outcome variable: developed in-hospital complications? No/Yes. AIS, American Spinal Injury Association impairment scale; Four W-MVA, four-wheel motorized vehicle accidents.

## Data Availability

The data used in this study are available from the corresponding author upon reasonable request.

## Abbreviations

The following abbreviations are used in this manuscript:

TSI	Traumatic spinal injury
OR	Odds ratio
CI	Confidence interval
AIS	American Spinal Injury Association Impairment Scale
ICU	Intensive care unit
TSCI	Traumatic spinal cord injury
KKUH	King Khalid University Hospital
STROBE	Strengthening the Reporting of Observational Studies in Epidemiology
EMR	Electronic medical record
CT	Computed tomography
MRI	Magnetic resonance imaging
Four W-MVA	Four-wheel motorized vehicle accident
Two W-MVA	Two-wheel motorized vehicle accident
IQR	Interquartile range
